# Blood gas levels, cardiovascular strain and cognitive performance during surgical mask and filtering face piece application

**DOI:** 10.1038/s41598-022-13711-2

**Published:** 2022-06-11

**Authors:** Katharina Grimm, Daniel Niederer, Albert Nienhaus, David A. Groneberg, Tobias Engeroff

**Affiliations:** 1 Division Health and Performance, Institute of Occupational, Social and Environmental Medicine, Goethe University Frankfurt, Theodor-Stern-Kai 7, Building 9B, 60590 Frankfurt am Main, Germany; 2 Institute of Occupational, Social and Environmental Medicine, Goethe University Frankfurt, Frankfurt am Main, Germany; 3 Department of Sports Medicine and Exercise Physiology, Institute of Sports Sciences, Goethe University Frankfurt, Frankfurt am Main, Germany; 4 Institute for Health Service Research in Dermatology and Nursing, University Medical Center Hamburg-Eppendorf, Hamburg, Germany

**Keywords:** Clinical trials, Randomized controlled trials

## Abstract

Mask induced airway resistance and carbon dioxide rebreathing is discussed to impact gas exchange and to induce discomfort and impairments in cognitive performance. N = 23 healthy humans (13 females, 10 males; 23.5 ± 2.1 years) participated in this randomized crossover trial (3 arms, 48-h washout periods). During interventions participants wore either a surgical face mask (SM), a filtering face piece (FFP2) or no mask (NM). Interventions included a 20-min siting period and 20 min steady state cycling on an ergometer at 77% of the maximal heart rate (HR). Hemodynamic data (HR, blood pressure), metabolic outcomes (pulse derived oxygen saturation, capillary carbon dioxide (pCO_2_), and oxygen partial pressure (pO_2_), lactate, pH, base excess), subjective response (ability to concentrate, arousal, perceived exertion) and cognitive performance (Stroop Test) were assessed. Compared to NM, both masks increased pCO_2_ (NM 31.9 ± 3.3 mmHg, SM = 35.2 ± 4.0 mmHg, FFP2 = 34.5 ± 3.8 mmHg, F = 12.670, p < 0.001) and decreased pH (NM = 7.42 ± 0.03, SM = 7.39 ± 0.03, FFP2 = 7.39 ± 0.04, F = 11.4, p < 0.001) during exercise. The FFP2 increased blood pressure during exercise (NM = 158 ± 15 mmHg, SM = 159 ± 16 mmHg, FFP2 = 162 ± 17 mmHg, F = 3.21, p = 0.050), the SM increased HR during sitting (NM = 70 ± 8 bpm, SM = 74 ± 8 bpm, FFP2 = 73 ± 8 bpm, F = 4.70, p = 0.014). No mask showed any comparative effect on other hemodynamic, metabolic, subjective, or cognitive outcomes. Mask wearing leads to slightly increased cardiovascular stress and elevated carbon dioxide levels during exercise but did not affect cognitive performance or wellbeing.

## Introduction

During the Covid-19 pandemic, mouth and nose protection against droplets and aerosols is ubiquitously applied. Due to their comparable high effectiveness in filtering particle emission^1^ and their potential superiority compared to cloth masks^2^, filtering face pieces type 2 (FFP2, comparable features to N95 respirators) and surgical mask are frequently recommended. Although limiting the risk of airborne infection is of utmost importance, potential negative effects of mask induced adaptations in respiratory function need to be further evaluated. Preliminary evidence indicates that masks might alter pulmonary function by increasing breathing resistance^3^ and the re-inhalation of exhaled air which is trapped between face and mask^4^. Increased breathing resistance can lead to a decrease in pulmonary function (breathing frequency, tidal volume and ventilation)^5,6^. The re-inhaled air is reported to contain less oxygen (17%) and more carbon dioxide (3.0%)^7^. The meta-analysis of our workgroup was able to detect significantly lower values for maximal oxygen uptake and pulse derived oxygen saturation during exhaustive exercise when a surgical mask or FFP2/N95 was applied^5^. Therefore, we hypothesize, that exacerbating the ability to breath by mask wearing during strenuous activities leads to limitations in oxygen uptake. The second currently available meta-analysis pooled the data for oxygen related outcomes (oxygen uptake, tissue oxygenation index, arterial-venous oxygen content difference) and reported no effect of masks during steady state exercise and maximal efforts^6^. Overall, these findings indicate that mask-induced breathing resistance and subsequent alterations of pulmonary function might limit gas exchange only during exhaustive physical activities.

An effect of mask wearing on carbon dioxide elimination is also still open to debate: randomized controlled studies found no effects of masks on blood oxygenation and carbon dioxide levels during rest^8^ or exhaustive exercise^9^. Since these studies did not match the maximal workload during exercise with and without masks and assessed metabolic data at exercise cessation, it remains unclear if steady state exercise with comparable workload leads to higher carbon dioxide or lower oxygen levels when a mask is applied. Furthermore, it is unknown if sex-dependent differences in structural elements of the aerobic energy system, body size and muscle mass^10^ might influence the effect of mask application.

Generally, effects of mask wearing during maximal efforts such as graded exercise tests until volitional exhaustion are well described^3,9,11–14^. On contrary, the effects of mask wearing during steady state exercise, mirroring situations of daily living (exercise training, physical labour and leisure time activities), are rare. So far only one randomized controlled trial analysed the effects of surgical mask application during continuous exercise on gas exchange and reported decreased breathing frequency, ventilation, oxygen uptake and carbon dioxide exhalation whereas heart rate (HR), blood pressure and cardiac output were higher when a surgical mask was applied^3^. Since this study did not apply blood gas analysis, it is unclear which changes in metabolism are linked to the reported reduction in pulmonary performance.

Another point which needs to be addressed by future studies is the clinical effect of changes in pulmonary function or gas exchange. Although hypercapnia (arterial carbon dioxide partial pressure > 45 mmHg) or hypoxia (arterial oxygen partial pressure < 80 mmHg) through mask wearing is highly unlikely^5^, self-report complaints, including headache or impaired cognitive performance, could be associated with even slightly elevated carbon dioxide concentrations^15^. Furthermore, some studies speculate that discomfort during mask wearing^9^ and respiratory fatigue^16^ might impair work capacity or lead to premature fatigue during cognitive or physical activity. Some authors even hypothesize that mask application might result in headache, impaired cognition^17^, or in cardiac or renal overload^18^ without providing clear evidence. Contrastingly, no detrimental reductions in well-being and performance^19^ and even positive effects of mask wearing during exercise on cognitive function^20^ were reported by early RCTs. It is of utmost importance to further evaluate these effects in order to prove that mask application during daily living does not lead to detrimental health consequences.

To address the gap of knowledge regarding the impact of currently recommended mask types (surgical mask, FFP2/N95) on gas exchange and resulting symptoms, we conducted an experimental study on the effects of rest (sitting) and exercise with wearing a FFP2/N95 or surgical masks on relevant hemodynamic (HR and blood pressure) and invasive metabolic outcomes (pulse derived oxygen saturation, capillary pCO_2_ and pO_2_, lactate, pH, base excess), subjective complaints (ability to concentrate, arousal) and cognitive performance (attention and executive function).

We hypothesize that (1) the FFP2/N95 respirators have a more pronounced effect on hemodynamic and metabolic outcomes when compared to surgical masks and no mask wearing during exercise and sitting. We further hypothesize, that (2) FFP2/N95 and surgical mask wearing during exercise or rest has an effect on subjective complaints and cognitive function.

## Methods

### Study design

This study has a randomized counterbalanced cross-over design and is approved by the ethics committee of the Department of Psychology and Sports Sciences of the Goethe University (2021-12, approved 18/04/2021). The trial was registered a priori (German Register for Clinical Trials, DRKS-ID: DRKS00024044, date of registration 10/08/2021). The trial was conducted in accordance with the ethical standards set down by the declaration of Helsinki with its recent modification of 2013 (Fortaleza)^21^.

### Participants

Recruitment was done in an academic university clinic in Germany. Eligibility criteria included being between 18 and 65 years of age with no (medical or psychosocial) contraindication against vigorous physical activity. Exclusion criteria were the intake of psychoactive substances (by self-declaration), cardiovascular-, pulmonary-, renal-, neurological-, or mental diseases, advanced degenerative musculoskeletal disorder and not completely healed sporting injury (that affect subjective quality of life or physical performance during bicycle ergometry).

Sample size calculations were performed based on an earlier study assessing CO2 kinetics during steady state exercise with a surgical face mask^3^. A calculation based on an effect size of 0.3 a significance level of 5% and an 80% power resulted in a sample size of at least 20 participants adopting a crossover design. Calculating with a drop-out rate of 15%, a total number of 23 participants were included in this study.

**Figure 1:**
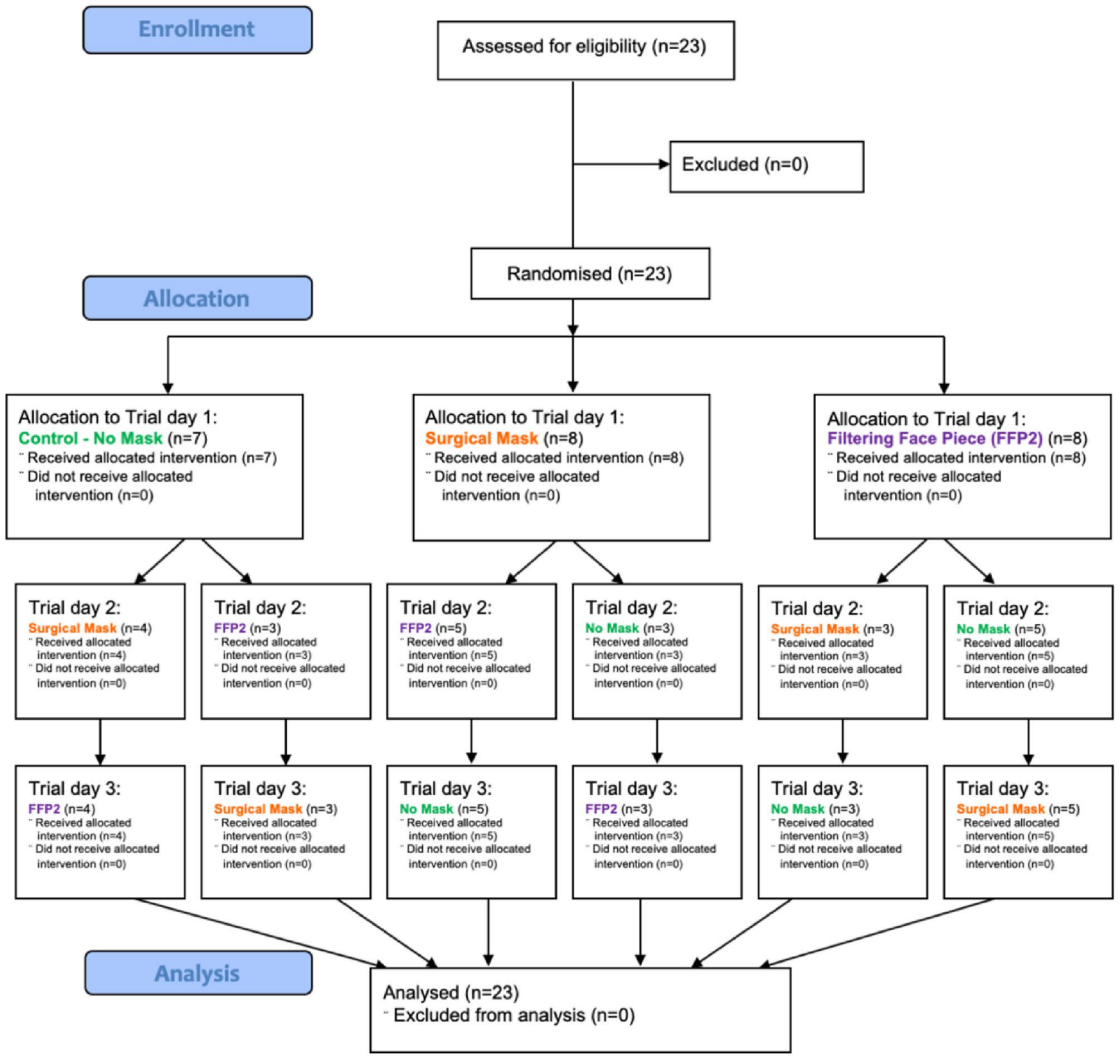
CONSORT flow diagram of the protocol procedures. Twenty-three participants were assigned to either the surgical face mask, the FFP2 mask or the unmasked condition by randomisation, followed by crossover to the other conditions. *NM* no mask, *SM* surgical mask, *FFP2* Filtering Face Piece Type 2, *CONSORT 2010* Consolidated Standards of Reporting.

Before study participation, participants were informed on voluntary participation and signed a written informed consent. Twenty-three volunteers (10 men, 13 women, 0 non-binary, age (mean ± SD): 23.5 ± 2.1 years) participated. Eligibility, exclusion and randomization scheme of the protocol is shown in the flow diagram in Fig. 1.

NM no mask, SM surgical mask, FFP2 Filtering Face Piece Type 2. CONSORT 2010, Consolidated Standards of Reporting.

### Interventions

Participants had to avoid vigorous physical activities in the 48 h preceding each test and to maintain their habitual diet behaviour during the timeframe of all three interventions. Furthermore, participants were requested not to take any food or drinks (except for water) in the period of 2 h prior to each examination.

All participants performed three different sessions at three different trial days (each separated by a minimum of 48 h and a maximum of 7 days). During each trial day, all participants once wore a surgical face mask, once a FFP2 mask (FFP2), and once no mask (NM) as manipulations. The manipulation order was counterbalanced and randomized (simple balanced randomization). As surgical mask, we used a 3-layer surgical mouth-nose protection (McAirlaid’s Vliesstoffe GmbH, Steinfurt, Germany). Both the surgical mask and the FFP2 (Ara Macao GmbH, Graben-Neudorf, Germany) were with ear loops and are available in public drugstores.

Each session at each trial day consisted of a 20-min sitting phase and a 20-min steady state bicycle ergometer exercise at vigorous intensity (77% maximal HR)^22^. Each session was performed at a comparable time of the day (± 2 h) and at days with comparable routines (i.e. working days). Order allocation was done blinded, the participants were blinded to the respective manipulation until the beginning of each intervention.

At each session, participants first rested for 20 min in a seated position without wearing a mask. The 20-min sitting phase followed; on a chair in reclined position. Here, the participants were allowed to read or use electronic devices. Between the sitting and ergometer intervention, there was a second 20-min resting period in which the participants were not manipulated (i.e. did not wear a mask). Afterwards, the bicycle exercise was performed, on an ergometer (Optibike med, ergoline GmbH, Bitz, Germany), at a constant cadence of 50–80 revolutions per minute (rpm). The intensity was set at 77% of the a priori estimated individual maximal HR during the first exercise manipulation^23^. Workload during interventions was 1.7 ± 0.4 Watt per kilogram bodyweight. During exercise, HR and perceived exertion were assessed each 3 min to control exercise intensity. Perceived exertion was assessed using a 15-point Borg Scale ranging from “very very light” (lowest rating 6) to “very very hard” (highest rating 20). Exercise should be perceived within the range of “somewhat hard” (rating 13) and “hard” (rating 16). Heart rate (in beats per minute, bpm) was monitored with a finger clip pulse oximeter (Nonin 8000SX, Plymouth, USA). Ergometer exercise started with a 2-min-long warm-up and resistance was increased stepwise until participants reached target HR and exertion values. Bicycle resistance was matched for all three interventions.

### Outcomes

#### Baseline assessments

Baseline examination took place before the first intervention. Assessments include standard anthropometrical values, educational status (school and study years), habitual physical activity and sedentary behaviour (International Physical Activity Questionnaire IPAQ; data analysed as metabolic equivalent of task hours METh)^24,25^. Subsequently, baseline HR and pulse derived oxygen saturation (SpO_2_ in percent) were assessed with a finger-clip sensor and systolic and diastolic blood pressure (millimetres mercury mmHg) was measured manually by a physician with an upper-arm cuff (MDF instruments, Germany) and a stethoscope (Littmann Classic, 3M, Neuss, Germany) (Riva-Rocci).

Baseline data for cognitive performance was assessed using a computerized version of a Stroop Test^26^. Participants familiarized themselves with the test by performing multiple trials in a standardized procedure prior to baseline assessment. Outcomes for cognitive performance were time to completion of five runs of a congruent test condition (OFF-Time in seconds), during which the participants were instructed to name the colour in which symbols were displayed on the tablet, and 5 runs of an incongruent test condition (ON-Time in seconds), during which colour words (i.e. the word “red”) were printed in a different colour (i.e. the word “red” printed in the colour blue) and participants should name the colour in which the word was printed (in our example “blue”).

For metabolic outcomes, capillary blood (100 µl) was drawn from an earlobe of the participant for blood gas analysis (epoc^®^ Blood Analysis System, Epocal Inc., Ottawa, Ontario, Canada). Outcomes included pH, carbon dioxide partial pressure (pCO_2_ in millimetres mercury, mmHg), oxygen partial pressure (pO_2_ in mm/Hg), lactate (mmol per litre, mmol/l) and base excess (BE in mmHg).

#### Assessments during interventions

On all three trial days HR, SpO_2_ and cognitive performance were assessed prior to the first manipulation (after the initial sitting phase). During sitting and exercise, HR was measured every 3 min. Within the last 5 min of the sitting intervention and the bicycle intervention, SpO_2_, subjective affective response (11-point Feeling Scale)^27^ and blood pressure were measured. During the last minute of the sitting and bicycle intervention, capillary blood samples were taken from an earlobe and were immediately analysed. After both, the sitting and the cycling intervention, participants completed cognitive performance testing and rated their subjective arousal (6 point scale) and ability to concentrate (visual analogue scale) via standardized scales^27^.

### Data analysis and statistics

We applied Microsoft Excel 2010 for data processing, Jamovi (Version 2.0.0) for data analysis, and Prism (Version 9) for data presentation. Descriptive data were reported as means and standard deviations (baseline values and post intervention values) or 95% confidence intervals (post intervention values and pre to post intervention changes).

Repeated measures analyses of variance (rmANOVA) were applied to analyse the effects of the manipulations (i.e. mask wearing: SMsurgical mask versus FFP2 versus NM) on the assessed values of all outcomes except the cognitive ones (here, as they were assessed pre- and post intervention, they were processed as change scores). In case of significant rmANOVA effects of manipulations, analysis of covariance (rmANCOVA) was applied. Co-variates were baseline values of outcomes and potential confounders (sex, age, weight, height, body mass index, physical activity, sedentary behaviour and baseline values of outcomes). We included rmANCOVA results in a supplementary file. We considered p ≤ 0.05 as statistically significant and applied a Bonferroni correction for multiple comparisons and post-hoc-analyses for the detailed between-manipulation-comparisons.

### Ethical approval

The study design was approved by the ethics committee of the Department of Psychology and Sports Sciences of the Goethe University (2021-12, Approved 2021/04/18).

## Results

### Demographic and baseline data

All participants completed the study protocol without any adverse event. Anthropometric and physical activity data as well as baseline characteristics for hemodynamic, metabolic and cognitive parameters are shown in Table 1. Weight, height, systolic blood pressure, diastolic blood pressure, pCO_2_ and base excess were lower in females compared to males. Baseline HR, blood oxygen saturation and pH values were higher in female participants at baseline.

**Table 1:** Baseline demographics and clinical characteristics.

Outcome/dimension	Unit	Participants			T-test
		Total	Males	Females	T-value, P-value df = 21
		Mean ± SD			
Age	years	23.5 ± 2.1	23.8 ± 2.7	23.2 ± 1.6	0.627, 0.538
Weight	kg	69.8 ± 16.6	81.7 ± 17.2	60.7 ± 8.8	3.827, < 0.001*
Height	cm	172 ± 10	181 ± 6	165 ± 5	7.381, < 0.001*
Body mass index	kg/m^2^	23.3 ± 3.5	24.8 ± 4.3	22.2 ± 2.4	1.854, 0.078
**Physical activity via IPAQ**					
Vigorous physical activity	min/week	75.4 ± 59.9	105.0 ± 65.2	52.7 ± 45.8	2.264, 0.034*
Moderate physical activity	min/week	73.0 ± 64.7	61.0 ± 64.1	82.3 ± 66.2	− 0.776, 0.447
Walking	min/week	124.6 ± 88.0	133.0 ± 82.8	118.1 ± 94.7	0.395, 0.697
Sedentary behaviour	min/day	391.3 ± 184.4	372.0 ± 132.1	406.2 ± 220.7	− 0.432, 0.670
Total physical activity	MET-min/week	1306.7 ± 674.0	1522.9 ± 692.9	1140.4 ± 635.4	1.376, 0.183
**Hemodynamic parameters**					
HR	bpm	79 ± 9	74 ± 9	83 ± 7	− 2.414, 0.025*
SBP	mmHg	123 ± 11	132 ± 9	117 ± 9	3.949, < 0.001*
DBP	mmHg	81 ± 8	87 ± 7	76 ± 5	4.387, < 0.001*
**Metabolic parameters**					
Pulse derived blood oxygen saturation	%	96.8 ± 1.0	96.2 ± 0.8	97.2 ± 0.9	− 2.816, 0.010*
pH		7.45 ± 0.02	7.44 ± 0.01	7.45 ± 0.02	− 2.639, 0.015*
pCO_2_	mmHg	34.53 ± 2.91	36.80 ± 2.29	32.78 ± 1.99	4.503, < 0.001*
pO_2_	mmHg	85.05 ± 16.18	84.51 ± 7.09	85.47 ± 21.02	− 0.138, 0.892
Lactate	mmol/l	1.30 ± 0.53	1.38 ± 0.60	1.24 ± 0.49	0.606, 0.551
Base excess	mmol/l	0.24 ± 1.32	0.99 ± 0.90	− 0.33 ± 1.33	2.690, 0.014*
**Cognitive parameters**					
School and study years	years	16.5 ± 0.9	16.4 ± 0.6	16.6 ± 1.1	− 0.576, 0.571
Stroop OFF-Time	sec	53.6 ± 6.3	52.6 ± 7.8	54.5 ± 5.1	− 0.715, 0.483
Stroop ON-Time	sec	57.4 ± 7.8	55.4 ± 9.7	58.9 ± 5.8	− 1.049, 0.306

Significant differences between sexes are indicated with an asterisk (*).

*HR* heart rate, *SBP* systolic blood pressure, *DBP* diastolic blood pressure, *pCO*_*2*_ partial pressure of carbon dioxide, *pO*_*2*_ partial pressure of oxygen, *MET* metabolic equivalent of task, *min* minute*, bpm* beats per minute, *sec* seconds.

*Independent Samples T-Test.

### Hemodynamic and metabolic data

**Figure 2:**
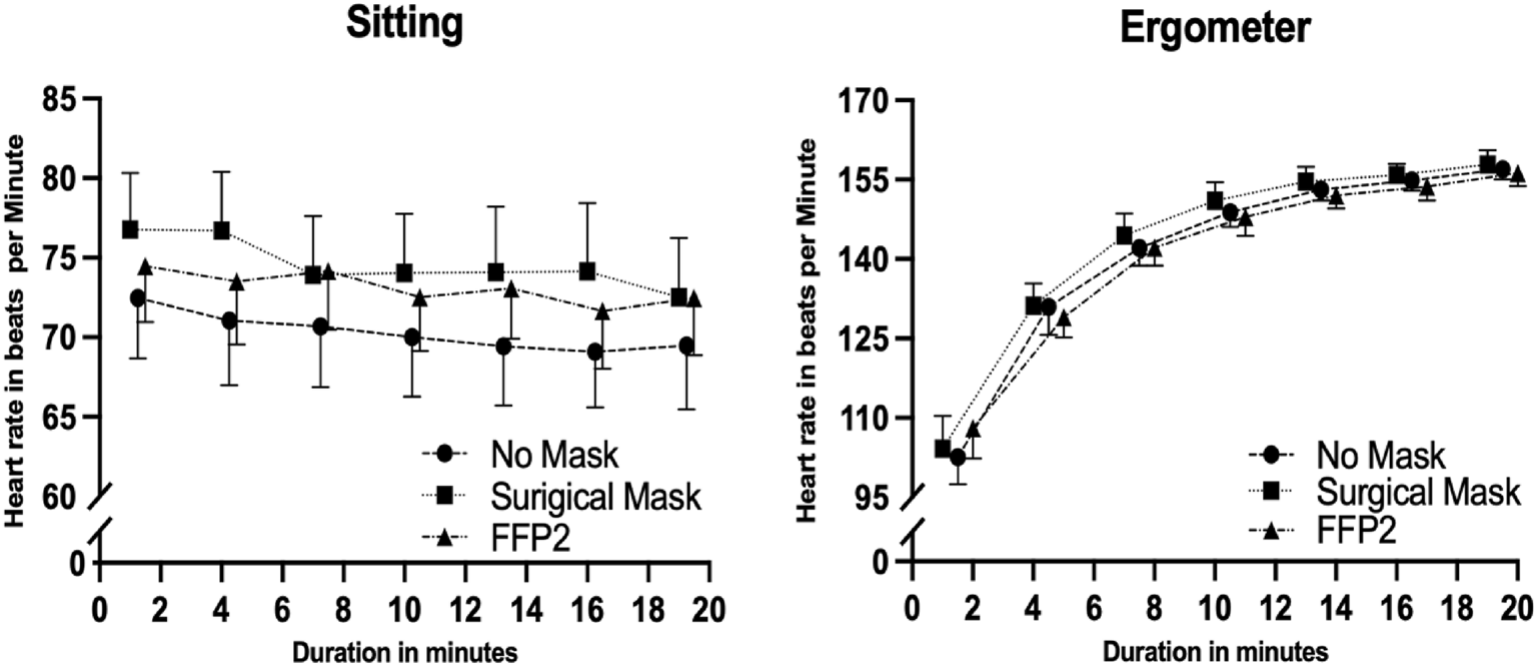
Mean values and 95% confidence intervals of heart rate data of all assessed time points during the sitting and the exercise condition with no mask, a surgical mask and a filtering face piece type 2 (FFP2).

The results of the between-manipulations-comparisons for the hemodynamic and metabolic data of the sitting phase are depicted in Table 2. A time effect (between manipulation effect) for mean HR values occurred. Mean HR was significantly lower in surgical mask mask condition compared to the no mask condition. Figure 2 shows the 95% confidence intervals of heart rate data for all assessed time points during the sitting and the exercise condition. The analyses of covariance revealed no effects of potential confounders (sex, age, weight, height, BMI, educational status, physical activity, sedentary behaviour and baseline values of outcomes). Manipulation (mask or no mask application) had no effect on any other haemodynamic or metabolic parameter during sitting.

**Table 2:** Results of hemodynamic, metabolic, subjective and performance outcomes during the sitting and bicycle ergometer intervention with no mask (NM), surgical mask (SM) or filtering face piece (FFP2) application.

Outcome	Unit	Manipulation			ANOVA time effect, F value, p value, effect size eta squared (η^2^)	Post-hoc comparisons p-values		
		No mask (NM)	Surgical mask (SM)	FFP2				
						NM vs. SM	NM vs. FFP2	SM vs. FFP2
**Sitting**								
Hemodynamic parameters								
HR	bpm	70 ± 8	74 ± 8	73 ± 8	4.70, 0.014*, 0.047	0.009	0.236	0.951
SBP	mmHg	119 ± 9	119 ± 10	119 ± 9	0.120, 0.887			
DBP	mmHg	79 ± 7	78 ± 7	79 ± 8	1.21, 0.309			
Metabolic parameters								
SpO_2_	%	97.2 ± 1.0	96.8 ± 0.9	97.0 ± 1.0	2.07, 0.193			
pH		7.44 ± 0.02	7.44 ± 0.03	7.44 ± 0.02	0.168, 0.846			
pCO_2_	mmHg	34.6 ± 3.16	35.3 ± 2.99	35.5 ± 3.42	1.86, 0.168			
PO_2_	mmHg	84.4 ± 7.01	84.0 ± 5.38	84.2 ± 6.21	0.0227, 0.978			
Lactate	mmol/l	1.24 ± 0.54	1.11 ± 0.43	1.11 ± 0.36	1.48, 0.238			
BE	mmol/l	− 0.04 ± 1.22	0.35 ± 1.42	0.21 ± 1.14	0.845, 0.437			
Subjective parameters								
Feeling scale		3.2 ± 1.5	2.8 ± 1.7	3.3 ± 1.7	0.906, 0.310			
Arousal		2.8 ± 1.3	2.6 ± 1.1	2.8 ± 1.1	0.304, 0.739			
Ability to concentrate		5.4 ± 1.9	5.6 ± 1.5	5.6 ± 1.6	0.459, 0.635			
**Exercise**								
Performance parameters								
Watt	W	118 ± 35	119 ± 35	118 ± 33	0.349, 0.707			
Relative Watt	W/kg	1.7 ± 0.4	1.7 ± 0.4	1.7 ± 0.3	0.432, 0.652			
Hemodynamic parameters								
HR	bpm	141 ± 5	143 ± 7	141 ± 6	0.725, 0.490			
SBP	mmHg	158 ± 15	159 ± 16	162 ± 17	3.21, 0.050*, 0.014	1.000	0.044	0.329
DBP	mmHg	75 ± 8	74 ± 6	75 ± 8	0.185, 0.832			
Metabolic parameters								
SpO_2_	%	95.6 ± 1.0	95.4 ± 1.2	95.4 ± 1.1	0.312, 0.734			
pH		7.42 ± 0.03	7.39 ± 0.03	7.39 ± 0.04	11.4, < .001*, 0.136	< 0.001	0.002	0.874
pCO_2_	mmHg	31.9 ± 3.33	35.2 ± 4.00	34.5 ± 3.80	13.1, < .001*, 0.134	< 0.001	0.006	0.879
PO_2_	mmHg	80.9 ± 7.13	80.5 ± 14.1	81.8 ± 17.9	0.0575, 0.944			
Lactate	mmol/l	5.49 ± 2.56	5.15 ± 2.35	5.48 ± 2.14	0.995, 0.378			
BE	mmol/l	− 3.03 ± 1.80	− 2.78 ± 2.04	− 3.33 ± 1.95	1.72, 0.190			
Subjective parameters								
Feeling scale		2.4 ± 1.7	1.8 ± 1.6	2.3 ± 1.6	2.04, 0.144			
Perceived exertion	Borg scale	14.1 ± 0.8	14.3 ± 0.9	14.5 ± 0.9	2.00, 0.147			
Arousal		4.5 ± 1.0	4.4 ± 0.8	4.5 ± 0.9	0.295, 0.746			
Ability to concentrate		6.0 ± 1.6	5.9 ± 2.0	6.1 ± 1.6	0.166, 0.848			

Significant effects of mask condition are indicated with asterisks (*).

*HR* heart rate, *SBP* systolic blood pressure, *DBP* diastolic blood pressure, *SpO*_*2*_ pulse derived oxygen saturation, *pCO*_*2*_ partial pressure of carbon dioxide, *pO*_*2*_ partial pressure of oxygen, *BE* base excess*, bpm* beats per minute.

**Figure 3:**
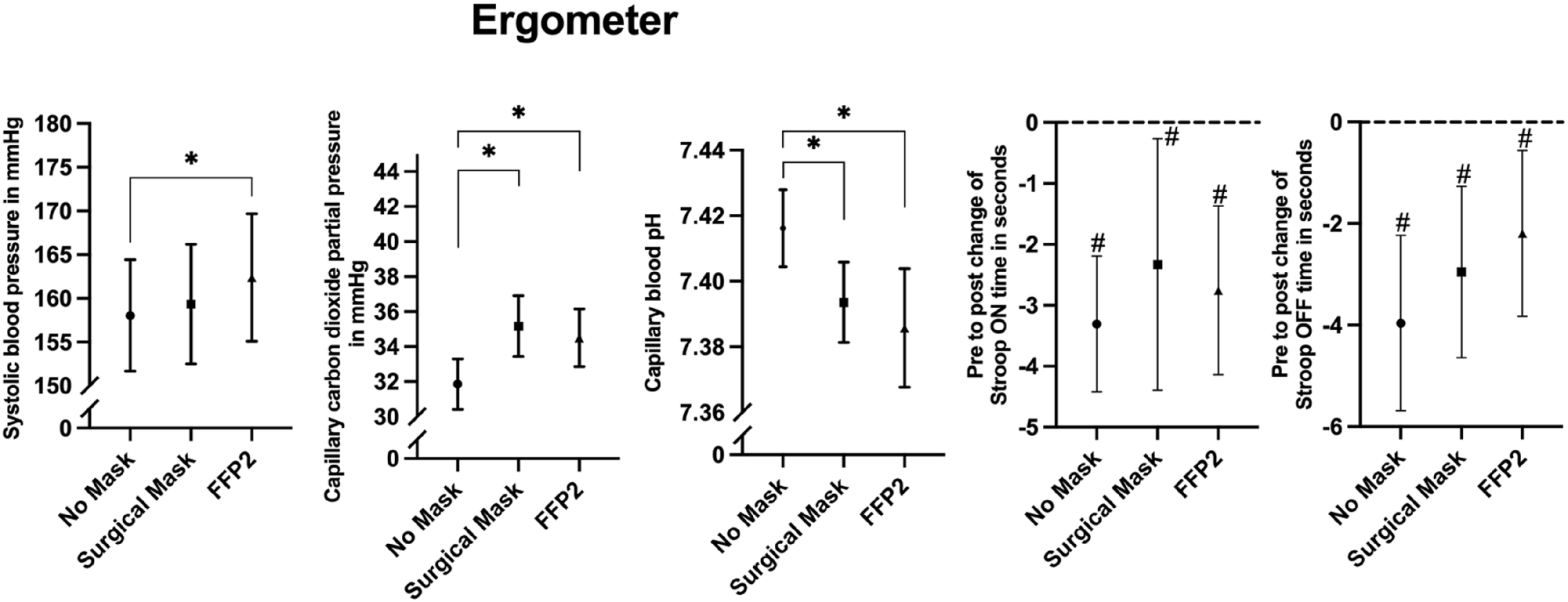
Mean values and 95% confidence intervals for systolic blood pressure, capillary blood carbon dioxide partial pressure and pH during exercise with no mask, a surgical mask or a filtering face piece type 2 (FFP2) and pre- to post exercise changes in cognitive attention (Stroop OFF-Time) and executive function (Stroop ON-Time) performance in seconds. Asterisks symbols (*) indicate significant time effects; Hash symbols (#) indicate significant pre to post exercise change in cognitive performance.

The results for hemodynamic and metabolic parameters during the exercise intervention are given in Table 2. The performance, thus the mean load on the bicycle ergometer, was not different between the unmasked and masked conditions. We found time effects on systolic blood pressure, pCO_2_ and pH during exercise. Systolic blood pressure was significantly higher when a FFP2 was applied compared to the no mask control condition. Both mask types showed an increasing effect on partial pressure of carbon dioxide during exercise compared to the no mask condition. The pH was lower during exercise when a surgical mask or a FFP2 was applied compared to the no mask control. Figure 3 displays the corresponding means and 95% confidence intervals of systolic blood pressure, pCO_2_ and pH for the exercise conditions. The analyses of covariance (supplementary file) showed no impact of the potential confounders (sex, age, weight, height, BMI, educational status, physical activity, sedentary behaviour and baseline values of outcomes).

### Self-reported data including cognitive performance

Descriptive data and results for self-perceived arousal and ability to concentrate as well as for affective response to both interventions and perceived exertion during the bicycle ergometer intervention are indicated in Table 2. Descriptive data and results for cognitive performance are indicated in Table 3. The 95% confidence intervals of pre to post changes in cognitive performance (Fig. 3) indicate that exercising with and without a mask led to a positive effect on cognitive performance. Cognitive performance remained unchanged after sitting. Mask application during sitting and exercising showed no effect on cognitive performance. Likewise, self-perceived arousal and ability to concentrate ratings were not affected by mask wearing during rest or exercise. Mask wearing also had no effect on affective response to the sitting and cycling intervention. The subjectively perceived exertion on the bicycle ergometer was not significantly different between the mask wearing and the no mask conditions.

**Table 3:** Pre intervention and pre to post intervention differences of cognitive attention (Stroop OFF-Time) and executive function (Stroop ON-Time) performance for the sitting and exercise intervention with no mask, surgical mask or filtering face piece type 2 (FFP2).

Outcome	Pre intervention values			Pre to post differences (post intervention values)			ANOVA
	No mask	Surgical mask	FFP2	No mask	Surgical mask	FFP2	F-value, p-value
**Sitting**							
OFF-Time	50.3 ± 7.3	49.4 ± 7.4	50.1 ± 6.7	− 1.2 ± 3.4 (49.0 ± 7.1)	− 0.3 ± 3.2 (49.1 ± 7.0)	0.5 ± 4.1 (50.6 ± 8.0)	1.24, 0.298 (1.38, 0.262)
ON-Time	53.1 ± 7.2	52.9 ± 7.7	54.4 ± 8.4	− 1.0 ± 3.0 (52.1 ± 7.4)	0.4 ± 5.0 (53.3 ± 8.0)	− 1.2 ± 4.1 (53.2 ± 8.2)	1.03, 0.366 (0.624, 0.540)
**Exercise**							
OFF-Time	50.3 ± 7.3	49.4 ± 7.4	50.1 ± 6.7	− 4.0 ± 4.0 (46.3 ± 6.2)	− 3.0 ± 3.9 (46.5 ± 6.8)	− 2.2 ± 3.8 (47.9 ± 7.0)	1.11, 0.337 (2.14, 0.130)
ON-Time	53.1 ± 7.2	52.9 ± 7.7	54.4 ± 8.4	− 3.3 ± 2.6 (49.8 ± 7.0)	− 2.3 ± 4.8 (50.6 ± 8.3)	− 2.8 ± 3.2 (51.6 ± 7.8)	0.408, 0.668 (1.61, 0.212)

Values are displayed as mean ± standard deviation.

## Discussion

Wearing a surgical mask or an FFP2 during exercises with vigorous intensity had a negative effect on capillary pCO_2_ and pH compared to a no mask control condition with matched workload. Our results thus confirm our first hypothesis. Although wearing surgical masks led to a less pronounced HR adaptation during 20 min sitting and wearing FFP2 during exercise led to a slightly more elevated systolic blood pressure, our data indicates no further detrimental changes in hemodynamic or metabolic parameters during exercise or sedentary behaviour (sitting). Affective responses to sitting and exercising were not influenced by mask wearing; hypothesis 2 must, thus, be rejected. Furthermore, self-perceived ability to concentrate and arousal after exercise were not affected by surgical masks and FFP2. Mouth nose protection during rest and exercise did not lead to detrimental changes in cognitive performance compared to the no mask control condition.

### Metabolic response to mask wearing

Our data leads to the assumption that either carbon dioxide production during intense muscular activity is increased or elimination via breathing is limited when a mask is worn. As a result, acid–base-balance is negatively affected by higher blood carbon dioxide levels which leads to a slight acidosis. Both hypotheses (increased production or decreased elimination of CO_2_) are in line with earlier findings, which indicate both, lower carbon dioxide exhalation and lower oxygen uptake during steady state exercise when a mask was applied^3^. Pooled data for graded and steady state exercises confirm effects of mask wearing during exercise on end tidal CO_2_ whereas oxygen saturation and exercise performance values seem not to be altered by mask wearing^6^. In line with these meta-analytic findings on non-invasive outcomes, capillary blood oxygen partial pressure was not affected by mask wearing during steady state exercise (this study) and graded exercise until volitional exhaustion^9^. Furthermore, ours and earlier data show that lactate does not accumulate to a larger extend during exercise, indicating that the rate of anaerobic metabolism is not altered by mask wearing^3,9^. Consequently, a limited carbon dioxide elimination via breathing during vigorous steady state exercise as main effect mechanism for our findings seems more likely.

### Hemodynamic response to mask wearing

We detected higher systolic blood pressure but comparable HR in a mixed population during exercise when a FFP2 is applied. These findings are in line with the study of Driver and colleagues who also report higher systolic blood pressure during high intensity exercise but not during lower intensity in a mixed population^28^. These changes in blood pressure could be based on higher peripheral vascular resistance or increased cardiac output. The intervention study of Lässing and colleagues show increased cardiac output during masked exercise with moderate intensity in male participants^3^. Overall, these findings indicate that cardiovascular performance is elevated during exercise when a mask is applied. It is likely that these adaptations are based on a response of sympathetic nerve activity to elevated CO2 levels and decreased pH^29^. Higher airway resistance could also trigger a cardiovascular response comparable to the mechanism which leads to pulsatile hypertension in sleep apnoea patients^30^. It is open to debate, if the higher resting HR during mask application in our study indicates a sufficient compensation strategy (increased cardiac output) for a less pronounced negative impact of mask-induced airway resistance and CO2 rebreathing on cardiopulmonary function during sitting. This would be one explanation for comparable metabolic responses during sitting with and without a mask.

### Impact of mask wearing on well-being and cognitive performance

Pooled data for incremental exercise until exhaustion indicate that mask wearing results in a more pronounced subjective response to exercise and higher perceived exertion^6,9^. Steady state exercise with moderate^3^ or vigorous intensity (our study) on the other hand seems to result in a comparable affective state with and without a mask.

Against anecdotical evidence^17^, we found no negative impact of mask wearing on the self-perceived ability to concentrate and cognitive performance and were able to confirm no detrimental consequences on well-being and performance^19^. Furthermore, the positive effects of exercise with moderate to vigorous intensity on cognitive performance^31–33^ seem not to be eliminated by mask wearing.

### Implications for exercise, training and everyday life

The application of FFP2 or surgical masks does not lead to clinically relevant detrimental effects during rest and exercise with an effort up to vigorous intensity. Consequently, steady state exercise or physical activity can be performed at comparable intensity in settings where masks are obligatory. Furthermore, office work performance or other cognitive demands seem not to be detrimentally affected and physically active or inactive commuting (cycling, walking, public transport) with a mask is unlikely to result in negative metabolic consequences.

Future studies need to evaluate if higher carbon dioxide levels, higher cardiac output or increased breathing resistance during exercise influence the metabolic response and other training induced adaptations. Based on meta-analytic findings, athletes might reach comparable maximal exercise performances with and without a mask^5,6^. It is however likely that mask induced changes in maximal oxygen uptake and pulmonary function alter the metabolic response to maximal exercise^5^.

## Limitations and future research

Future studies need to assess if physical activities with lower intensity lead to comparable effects on cardiac output and blood carbon dioxide levels. Based on our design we were not able to measure pulmonary performance. Although the application of rubber masks (to measure breathing components) is likely to alter the airway resistance and the volume of air trapped between face and surgical or FFP2/N95 masks, further studies might include both, blood gas analysis and spiroergometric measures to analyse the impact of mask wearing on carbon dioxide metabolism and pulmonary function. Furthermore, the objective or self-reported assessment of respiratory fatigue is of high interest.

## Conclusion

Face mask such as surgical masks and FFP2 induce small changes in pulmonary function, which, in turn, lead to slightly increased blood carbon dioxide levels during physical activity with high intensity. All carbon dioxide values were, yet, in a physiological range and did not affect cognitive performance and subjective wellbeing. Therefore, we found no evidence for detrimental health effects of mask application in settings without the option to maintain social distancing over a limited timeframe, such as commutes (cycling or walking) in public areas, public transport or car sharing. Cardiovascular performance seems to only be upregulated to a minor share during mask wearing. Consequently, healthy adults are able to physiologically compensate the impact of mouth and nose protections masks during rest and physical activities (i.e. exercise), if a metabolic steady state can be obtained. Further studies need to assess the effects of prolonged and repeated mask application especially in realistic settings (shared workspace, long distance public transport) including physical activity (i.e. physical labour in crowded areas) to further evaluate potential effects on well-being and metabolism.

## Supplementary Information

Supplementary Information.

## Data Availability

Data are available upon reasonable request per institutional policy.
