# Supplementary material for: Blood gas levels, cardiovascular strain and cognitive performance during surgical mask and filtering face piece application
Source: Sci Rep. 2022 Jun 11;12:9708. doi: 10.1038/s41598-022-13711-2 (PMC9188275; doi:10.1038/s41598-022-13711-2)
Supplement: Supplementary file 1 — Supplementary Information. [file 41598_2022_13711_MOESM1_ESM.docx]

| Outcome | Unit | Manipulation | | | rmANCOVA  time effect and  between subject effects of covariates (interaction effects) | ANOVA  F value,  p value |
| --- | --- | --- | --- | --- | --- | --- |
|  |  | No mask | Surgical mask | FFP2 |  |  |
| SITTING condition | | | | | | |
| Hemodynamic parameters | | | | | | |
| HR | bpm | 70±8 | 74±8 | 73±8 | Time effect | 4.70, 0.014* |
|  |  |  |  |  | Gender | 0.28, 0.603 |
|  |  |  |  |  | Age in years | 1.11, 0.311 |
|  |  |  |  |  | Weight in kilograms (kg) | 0.02, 0.881 |
|  |  |  |  |  | Height in centimetres (cm) | 0.01, 0.977 |
|  |  |  |  |  | BMI in kg/m^2^ | 0.05, 0.822 |
|  |  |  |  |  | Physical Activity in  MET-min/wk | 0.88, 0.365 |
|  |  |  |  |  | Sedentary time in min/wk | 0.01, 0.938 |
|  |  |  |  |  | Baseline HR | 29.74, 0.001*  (0.03, 0.971) |
| EXERCISE condition | | | | | | |
| Hemodynamic parameters | | | | | | |
| SBP | mmHg | 158±15 | 159±16 | 162±17 | Time effect | 3.21, 0.050* |
|  |  |  |  |  | Gender | 0.01, 0.974 |
|  |  |  |  |  | Age in years | 0.06, 0.811 |
|  |  |  |  |  | Weight in kilograms (kg) | 1.63, 0.223 |
|  |  |  |  |  | Height in centimetres (cm) | 3.54, 0.081 |
|  |  |  |  |  | BMI in kg/m^2^ | 2.09, 0.170 |
|  |  |  |  |  | Physical Activity in  MET-min/wk | 0.01, 0.981 |
|  |  |  |  |  | Sedentary time in min/wk | 0.03, 0.858 |
|  |  |  |  |  | Baseline SBP | 5.46, 0.035 |
| Metabolic parameters | | | | | | |
| pH |  | 7.42±0.03 | 7.39±0.03 | 7.39±0.04 | Time effect | 11.4, < .001* |
|  |  |  |  |  | Gender | 0.04, 0.850 |
|  |  |  |  |  | Age in years | 0.05, 0.832 |
|  |  |  |  |  | Weight in kilograms (kg) | 0.03, 0.866 |
|  |  |  |  |  | Height in centimetres (cm) | 0.01, 0.986 |
|  |  |  |  |  | BMI in kg/m^2^ | 0.01, 0.936 |
|  |  |  |  |  | Physical Activity in  MET-min/wk | 0.93, 0.352 |
|  |  |  |  |  | Sedentary time in min/wk | 0.01, 0.781 |
|  |  |  |  |  | Baseline pH | 0.84, 0.375 |
|  | | | | | | |
| pCO2 | mmHg | 31.9±3.33 | 35.2±4.00 | 34.5±3.80 | Time effect | 13.1, < .001* |
|  |  |  |  |  | Gender | 1.55, 0.233 |
|  |  |  |  |  | Age in years | 0.28, 0.608 |
|  |  |  |  |  | Weight in kilograms (kg) | 1.94, 0.185 |
|  |  |  |  |  | Height in centimetres (cm) | 0.67, 0.428 |
|  |  |  |  |  | BMI in kg/m^2^ | 2.23, 0.157 |
|  |  |  |  |  | Physical Activity in  MET-min/wk | 0.05, 0.814 |
|  |  |  |  |  | Sedentary time in min/wk | 0.20, 0.663 |
|  |  |  |  |  | Baseline pCO2 | 6.98, 0.019*  (0.66, 0.527) |
| Note: Significant effects of mask condition or between subject effects of covariates are indicated with asterisks (*).  *HR* heart rate, *SBP* systolic blood pressure, *pCO2* partial pressure of carbon dioxide, *bpm* beats per minute, *mmHg* millimetres mercury, *min* minutes, *wk* week | | | | | | |
